# Increased risk of periprosthetic joint infection after traumatic injury in joint revision patients

**DOI:** 10.1186/s42836-024-00235-5

**Published:** 2024-02-05

**Authors:** Hao Li, Qingyuan Zheng, Erlong Niu, Jiazheng Xu, Wei Chai, Chi Xu, Jun Fu, Libo Hao, Jiying Chen, Guoqiang Zhang

**Affiliations:** 1 Medical School of Chinese PLA, Beijing, 100853 China; 2 Department of Orthopedic Surgery, First Medical Center, Chinese PLA General Hospital, Beijing, 100853 China; 3 Senior Department of Orthopedics, Fourth Medical Center, Chinese PLA General Hospital, Beijing, 100048 China; 4 Department of Orthopedics, Fifth Medical Center, Chinese PLA General Hospital, Beijing, 100039 China

**Keywords:** Periprosthetic joint infection, Total joint arthroplasty, Revisions, Traumatic injury

## Abstract

**Background:**

Periprosthetic joint infection (PJI) is a serious complication after total joint arthroplasty (TJA). Although some risk factors of PJI were well studied, the association between trauma and PJI remains unknown in revision patients.

**Materials and methods:**

Between 2015 and 2018, a total of 71 patients with trauma history before revisions (trauma cohort) were propensity score matched (PSM) at a ratio of 1 to 5 with a control cohort of revision patients without a history of trauma. Then, the cumulative incidence rate of PJI within 3 years after operation between the two groups was compared. The secondary endpoints were aseptic revisions within 3 postoperative years, complications up to 30 postoperative days, and readmission up to 90 days. During a minimal 3-year follow-up, the survival was comparatively analyzed between the trauma cohort and the control cohort.

**Results:**

The cumulative incidence of PJI was 40.85% in patients with trauma history against 27.04% in the controls (*P* = 0.02). Correspondingly, the cumulative incidence of aseptic re-revisions was 12.68% in patients with trauma history compared with 5.07% in the control cohort (*P* = 0.028). Cox regression revealed that trauma history was a risk factor of PJI (HR, 1.533 [95%CI, (1.019,2.306)]; *P* = 0.04) and aseptic re-revisions (HR, 3.285 [95%CI, (1.790,6.028)]; *P* < 0.0001).

**Conclusions:**

Our study demonstrated that revision patients with trauma history carried a higher risk of PJI compared to those without trauma history. Moreover, after revisions, the trauma patients were still at higher risk for treatment failure due to PJI, periprosthetic joint fracture, and mechanical complications.

**Supplementary Information:**

The online version contains supplementary material available at 10.1186/s42836-024-00235-5.

## Background

Total knee arthroplasty and total hip arthroplasty are deemed well-established and successful procedures because they can relieve pain and improve the quality of life for patients with advanced joint diseases [1]. However, some complications occur after total joint arthroplasty (TJA), such as aseptic loosening, periprosthetic joint infection, and dislocation. Among them, periprosthetic joint infection (PJI) is one of the most disastrous post-TJA complications and is often indicative of unfavorable outcomes [1–3]. Although a higher risk of PJI in populations with obesity, rheumatoid arthritis (RA), post-traumatic arthritis, nutritional status, diabetes mellitus (DM), and multiple revisions are well-established, the data on patients with prior trauma before revisions have been scanty [1, 4–7].

The post-traumatic arthritis is associated with increased risks of subsequent surgical site infection and periprosthetic joint infection after joint arthroplasty, especially in patients previously having received surgeries and with retained implants [7–9]. However, the impact of trauma history before revisions on the post-revision outcomes was less clear. Since the risk of PJI is increased after TJA in post-traumatic arthritis patients, we believed that this might be true of revision patients with trauma history.

In this study, we hypothesized that the patients with a trauma history before revisions might be at a higher risk of PJI compared to those without. This study was designed to answer the questions: “Are revision patients with prior trauma history at an elevated risk for PJI before revisions?” and “Are they still at a higher risk for infectious and aseptic re-revisions 3 years after revisions?” This matched cohort study examined the influence of trauma history on the incidence of PJI.

## Materials and methods

### Trauma definition and data collection

Institutional Review Board approval was obtained before the commencement of this study. The study was conducted in a tertiary care orthopedic center. The medical data about trauma history were harvested by a thorough chart review of the revision cases admitted to our institution between 2015 and 2018. The “trauma” was defined as follows:

1. Trauma and periprosthetic joint fracture as identified by X-ray before revisions;
2. Wrench injury, tumble injury around the joint before revisions, but no traumatic wound dehiscence happened after trauma.

Wrench injury: the joint sustained a violent twist;

Tumble injury: the joint was hurt due to a sudden downfall from standing and the joint was hurt while no traumatic wound dehiscence happened after trauma.

### Inclusion criteria of trauma cohort

1. Patients who complained of trauma and closed periprosthetic joint fracture within 3 months before joint revisions.
2. Patients with closed periprosthetic joint fracture that was identified by X-ray before revisions.

### Exclusion criteria of trauma cohort

1. Open periprosthetic joint fracture before revisions;
2. Dehiscence happened after trauma before revisions
3. Open periprosthetic joint fracture or dehiscence after revisions

Once the subjects were identified, their charts were retrospectively reviewed to ascertain whether the patients had developed PJI, as defined by the 2014 MSIS criteria [10]. Besides, the following data were also collected: the type of revision surgeries, the culture results, the time interval between revision and end outcomes, the age, sex, body mass index (BMI), ASA scores, the joint involved, complications, the comorbidities, including liver, kidney, heart, lung diseases, diabetes, and inflammatory joint diseases.

### Study design and propensity score matching

**Fig. 1:**
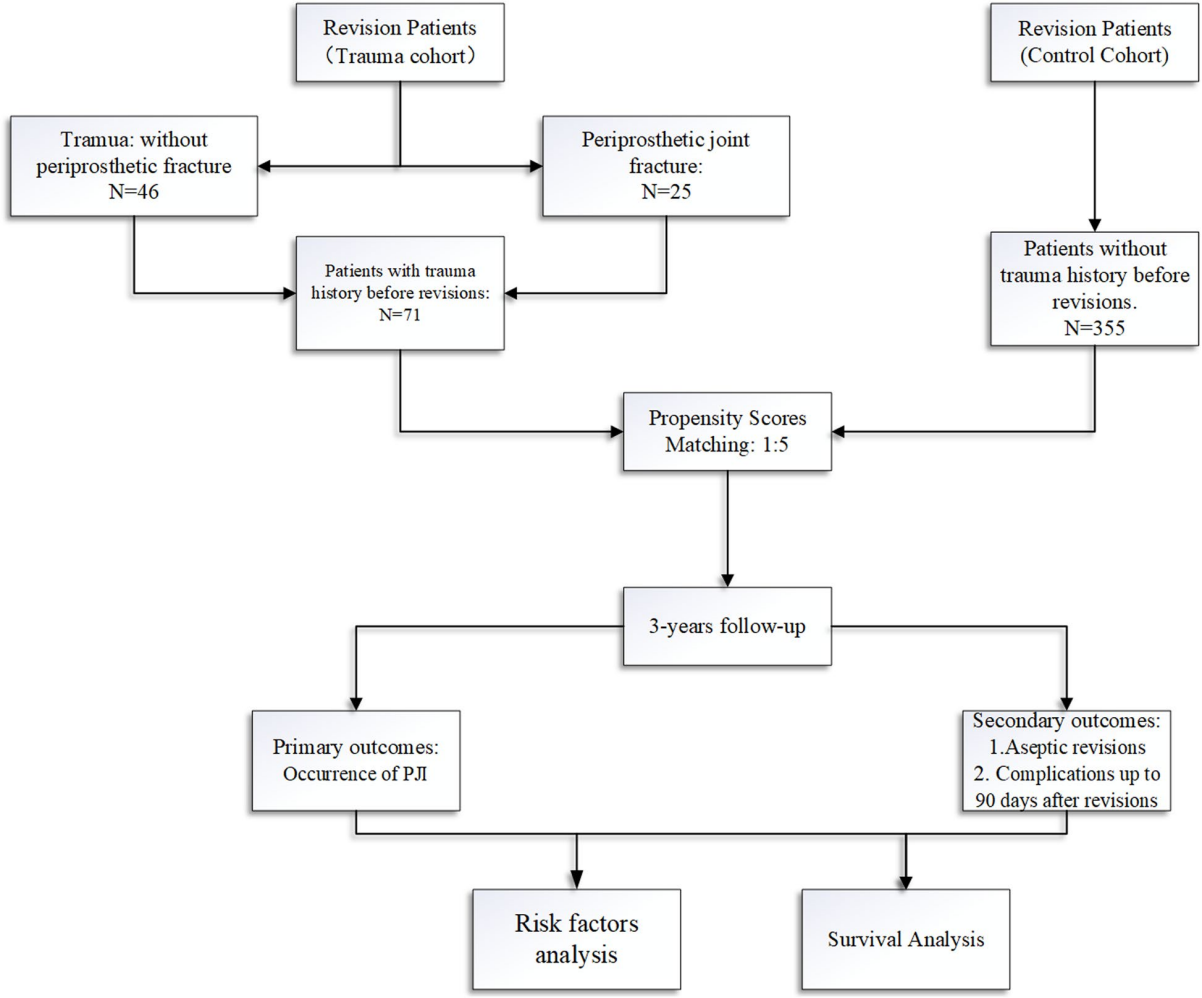
The study design and patients included in this study

Upon selection, a total of 71 patients with a trauma history were included as the trauma cohort. During the same period, we performed approximately 1,000 joint revisions for various reasons and the patients were potentially eligible for case matching because they were available for follow-up at a minimum of 3 years. The 71 patients in the trauma cohort (TKA: 28 cases and THA: 46 cases) were propensity score matched (PSM), at 1 to 5, with a control cohort of revision patients without a history of trauma. The PSM was based on age, sex, BMI, involved joint, ASA scores, months from the last TJA, and the presence of RA and DM. Then the baseline characteristics were compared between the trauma cohort and controls. The study design is summarizd in Fig. 1 and the PSM results are detailed in Additional file 1.

### Endpoints

In this study, we conducted a minimum of three years of follow-up and two endpoints were defined in the follow-up.

1. The primary end point was the existence of PJI before revisions and the occurrence of PJI within 3 years after operation.
2. The secondary end points were the occurrence of aseptic re-revisions within 3 years after operation, complications up to 30 days after revisions and readmission up to 90 days.

The null hypothesis was that there existed no difference between the trauma cohort and control cohort.

### Postoperative management

The drainage tubes were placed for > 3 days until the drainage volume <  = 50 mL/day. Antibiotics were used for two weeks after revisions. The antibiotics administered were changed according to the intraoperative culture results. If intraoperative culture yielded negative results, ceftriaxone was given postoperatively.

### Statistical analysis

The baseline characteristics of the patients were described as continuous data and dichotomous data. The continuous data were presented as means or medians. The *t*-test was adapted to compare these data if continuous variables followed a pattern of normal distribution. Otherwise, the rank-sum test was utilized. Dichotomous data were expressed as frequencies and percentages. Then, these data were compared by using chi-squared test or Fisher exact test. Three univariate regressions were performed with Cox regression model to identify the hazard ratio (HR) of trauma history: the dependent outcome was PJI in the first model, aseptic revision in the second model and the primary in combination the second outcome in the third model. The log-rank test was used to compare the survival rates between the two cohorts. In this study, the Log Rank Test Power Analysis was performed by using Lakatos method [11, 12]. The sample sizes included in this study had a power of 80%, at an alpha of 0.05 to identify a > 1.52 HR in the survival analysis. Statistical significance was defined if *P* < 0.05 and statistical analysis was performed on SPSS (IBM version: 22.0), R (Version: 4.1.1), and Excel (Microsoft version: 2018). Power analysis was performed on PASS 11.0.

## Results

### The demographic characteristics of patients included in this study

After selection, a total of 426 patients, involving 71 PJI patients and 355 aseptic patients, were included in this study. The median age in the PJI cohort and aseptic cohort was 65 and 63 years, respectively. And there were 34 and 205 females in the trauma cohort and control cohort, respectively. No significant differences were found in the ASA scores, involved joint, BMI and age between the two cohorts. The demographic features of patients included in this study are shown in Table 1. Moreover, the trauma history of patients in the trauma cohort is listed in Additional file 2: Table S1.

**Table 1:** The demographic features of patients recruited

	Trauma cohort *n* = 71	Control cohort *n* = 355	*P* values
**Sex**			
Female, *n* (%)	34, 47.9%	205, 57.7%	0.126
Males, *n* (%)	37, 52.1%	150, 42.3%	0.126
Age (year)**	65, (59,74)	63, (53,71)	0.095
**Body mass index (kg/m**^**2**^)**	24.22, (22.60, 27.18)	25.24 (22.89, 27.36)	0.339
**Involved joint**			
Hip, *n* (%)	43, 60.6%	207, 58.3%	0.725
Knee, *n* (%)	28, 39.4%	148, 41.7%	0.725
**ASA score**			
1, *n* (%)	1, 1.4%	4, 1.1%	0.386
2, *n* (%)	54, 76.1%	299, 84.2%	0.386
3, *n* (%)	15, 21.1%	50, 14.1%	0.386
4, *n* (%)	1, 1.4%	2, 0.6%	0.386
**PJI type**			
Early infection, *n* (%)^b^	8, 11.3%	6, 1.7%	< 0.0001*
Chronic infection, *n* (%)^c^	21, 29.6%	90, 25.3%	0.459
**Heart disease, *n* (%)**	8, 11.2%	27, 7.6%	0.305
**Kidney disease, *n* (%)**	0	0	1
**Smoking, *n* (%)**	12, 16.9%	43, 12.1%	0.468
**Drinking, *n* (%)**	12, 16.9%	42, 11.9%	0.385
**RA**, ***n*** **(%)**	5, 7%	31, 8.7%	0.815
**Revision-cause**			
PJI (septic revision), *n* (%)	29, 40.85%	96, 27.04%	0.02*
**DAIR**^a^, *n* (%)	9, 31.03%	33, 34.37%	0.739
1-stage revisions^a^, *n* (%)	7, 24.13%	28, 29.17%	0.597
2-stage revisions^a^, *n* (%)	13, 44.83%	35, 36.46%	0.417
Aseptic revision, *n* (%)	42, 59.15%	259, 72.96%	0.02*
**Re-revision-cause, *n* (%)**	17, 23.94%	27, 7.61%	< 0.0001*
PJI (septic re-revision), *n* (%)	8, 11.27%	9, 2.54%	0.003*
Aseptic re-revision, *n* (%)	9, 12.68%	18, 5.07%	0.028*

^*^P < 0.05

**The values were given as medians with the IQR (interquartile range) in the paratheses

^a^The values were given as cases and the constitution ratio

^b^Early infection: the first postoperative month/s < 3 weeks of symptoms

^c^Chronic infection: the first postoperative month/s > 4 weeks of symptoms. Items of bold type indicate variables

### The infection-associated makers between the trauma cohort and controls

In this study, the infection-associated markers were compared and the patients in the trauma cohort had relatively stronger inflammatory responses than those in the control cohort. The levels of CRP, plasma D-dimer, plasma fibrinogen, and the presence of sinus in the trauma cohort were significantly higher in the trauma cohort than in the control cohort. Besides, the synovial fluid WBC count and PMN% were also significantly higher in the trauma cohort than in the control cohort. The details about these markers are summarized in Table 2. In the trauma cohort, the most common cultured pathogens were *Staphylococcus spp.* (22 cases, 30.98%), followed by Gram-negative pathogens (3 cases, 4.23%) and *Streptococcus spp.* (2 cases, 2.82%). In the control cohort, the types of microorganisms showed similar pattern. The types of cultured pathogens in the trauma cohort and control cohort are shown in Table 3.

**Table 2:** The infection-associated markers in the trauma cohort and control cohort

	Trauma cohort *n* = 71	Control cohort *n* = 355	*P* values
ESR^a^ (mm/h)	18 (9, 39)	13 (6, 31)	0.081
CRP^a^ (mg/dL)	0.92 (0.21, 2.71)	0.33 (0.10, 1.29)	0.001**
Plasma D-dimer^a^ (μg/mL)	1.51 (0.75, 2.96)	0.98 (0.55, 1.82)	< 0.0001**
Plasma fibrinogen^a^ (g/L)	4.09 (3.11, 5.10)	3.54 (2.91, 4.92)	0.033**
Synovial fluid WBC Count^a^ (cells/μL)	1,530 (300, 7,690)	440 (63, 6,723)	0.014**
Synovial fluid PMN%^a^	80 (54, 93.5)	28.5 (10, 59)	< 0.0001**
Positive histological analysis (> 5 neutrophil/HP)	22, 31%	132, 37.2%	0.321
The presence of Sinus, *n* (%)	15, (21.1%)	26, (7.3%)	0.001**
Two identical cultures, *n* (%)	11, (15.49%)	59, (16.62%)	0.815
Positive culture, *n* (%)	27, (38.03%)	111, (31.27%)	0.266

^a^The values were given as medians (Second Quartile, Third Quartile)

^**^P < 0.05. Items of bold type indicate variables

**Table 3:** The types of pathogenic microorganisms in the two cohorts

Cultured Microorganisms	Trauma cohort *n* = 71	Control Cohort *n* = 355	*P* values
Staphylococcus spp.	22, 30.98%	69, 19.44%	0.030
Staphylococcus aureus	6, 8.45%	19, 5.35%	0.281
Coagulation-negative Staphylococcus	16, 22.54%	50, 14.08%	0.072
Enterococcus spp.	1, 1.41%	10, 2.82%	0.700
E. coli	2, 2.82%	7, 1.97%	1
Streptococcus spp.	2, 2.82%	7, 1.97%	1
Gram positive bacillus	2, 2.82%	4, 1.13%	0.268
Gram negative pathogens	3, 4.23%	15, 4.22%	0.773
Fungus	1, 1.41%	4, 1.13%	1
Polymicrobial culture results	4, 5.63%	11, 3.10%	0.497

### Survival analysis for the patients with or without trauma history before revisions

**Fig. 2:**
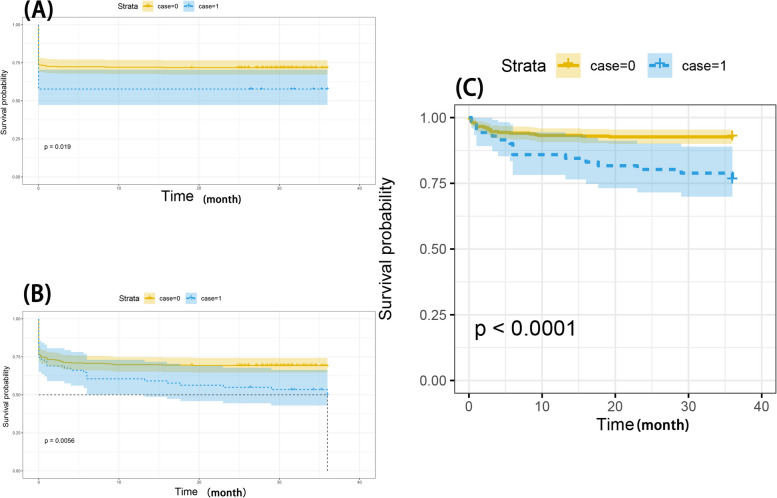
**A** Kaplan–Meier curve for PJI incidence; **B** Kaplan–Meier curve for PJI and aseptic re-revisions; **C** The Kaplan–Meier curve of aseptic re-revisions for PJI after revisions

To examine the impact of trauma history on the occurrence of PJI and aseptic re-revision, a survival analysis was performed. Firstly, the occurrence of PJI was designated the primary endpoint, and the patients in the control group were more likely to be free of PJI compared to their counterparts in the trauma cohort (the rate of PJI: 40.85% vs. 27.04%; *P* = 0.02) (Fig. 2A). Then the bivariate Cox regression for the trauma history was performed and PJI was found to be significantly associated with trauma history (HR, 1.533 [95%CI, (1.019, 2.306)]; *P* = 0.04) (Table 4). In the control cohort, patients (96 cases) who developed PJI was treated with DAIR (debridement, antibiotics, and implant retention) (33 cases, 34%), one-stage revisions (28 cases, 29%) or two-stage revisions (35 cases, 37%). In the trauma cohort, patients (29 cases) who developed PJI were treated with DAIR (debridement, antibiotics, and implant retention) (9 cases, 31%), one-stage revisions (7 cases, 24%) or two-stage revisions (13 cases, 45%). Secondly, patients with trauma history also showed a higher rate of aseptic re-revisions (12.68% vs. 5.07%; *P* = 0.028) (Table 1) after revisions. The most common cause of re-revision was periprosthetic joint fracture (3 cases, 35%), mechanical complications (2 cases, 24%), and dislocation (1 case, 12%) in the trauma cohort. In the control group, the most common re-revision cause was periprosthetic joint fracture (4 cases, 22.2%), dislocation (4 cases, 22.2%) and mechanical complications (3 cases, 16.67%). No statistical difference was detected between the two cohorts when these aseptic complications were compared separately, i.e., periprosthetic joint fracture (4.23% vs. 1.27%, *P* = 0.094), dislocation (1.41% vs. 1.13%; *P* = 0.6) and mechanical complications (2.82% vs. 0.85%; *P* = 0.195). When a Cox regression model was built, trauma history before revisions was associated with a 3.285-fold higher risk of aseptic re-revisions (HR, 3.285 [95%CI, (1.790, 6.028)]; *P* < 0.0001) (Table 4) (Fig. 2C). Finally, the primary outcomes (PJI) and the secondary outcomes (aseptic re-revisions) were combined as the endpoints. The log-rank test showed significant difference (*P* = 0.0056) when the 2 cumulative survival curves were compared, and the patients without trauma history were more likely to be free of complications after revisions compared to those in the trauma cohort. In combination, patients with trauma history before revisions were at a higher risk of PJI and aseptic re-revisions (HR, 1.979 [95%CI, (1.079, 3.630)]; *P* = 0.027) (Fig. 2C).

**Table 4:** Bivariate cox regression for the trauma history

Variable	Estimate	*P* value	HR^a^
The incidence of PJI	0.427	0.04	1.533 (1.019, 2.306)
The incidence of PJI and aseptic re-revision	0.683	0.027	1.979 (1.079, 3.630)
The incidence of aseptic re-revision	1.189	< 0.0001	3.285 (1.790, 6.028)

^a^The values were given as the hazard ratio for patients with trauma history with the 95%CI in the parentheses

## Discussion

PJI is a serious complication after total joint arthroplasty [1]. In recent years, the risk factors of PJI have been explored but the association between trauma and PJI still remains unknown. We hypothesized that patients with trauma history after TJA are at higher risk of PJI. To confirm this hypothesis, we conducted a retrospective study and found that TJA patients with trauma history were more likely to suffer from PJI after revisions than those without. A significant finding of our study was that trauma before joint revisions was a risk factor of periprosthetic joint infection after joint revisions. Therefore, for patients with trauma history, special caution should be exercised to avoid PJI. Moreover, we found that patients with trauma history were more likely to develop aseptic complications after revisions. These results suggest that patients with trauma history should be given special attention and preventive measures should be taken to obviate non-infectious complications after joint revisions.

**Fig. 3:**
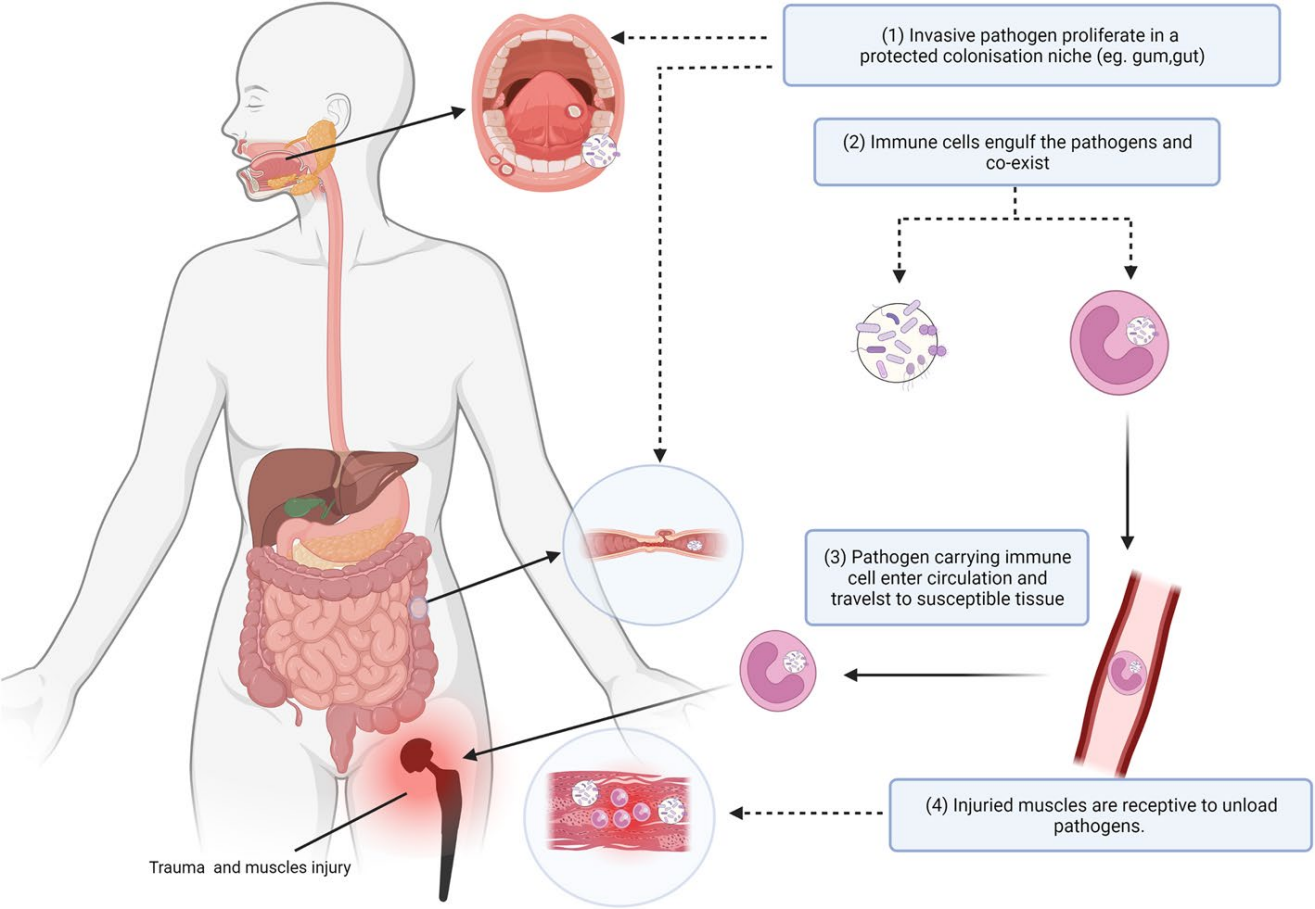
Trojan Horse hypothesis of trauma-associated periprosthetic joint infection. Schematic representation of the mechanisms by which pathogens from other sites cause periprosthetic infection

The patients with trauma history before revisions had a considerably higher risk of PJI. Some PJI risk factors such as BMI, the presence of diabetes, RA and smoking have been well established on the basis of previous studies but the prior trauma before revisions, as a risk factor, have been less studied [6]. Therefore, to know whether trauma history is a risk factor of PJI, we performed a retrospective cohort study to examine the association between the trauma and PJI after adjusting confounding factors by propensity score matching at 1:5. Our study suggested that the injured joint after total joint arthroplasty were more likely to be infected not only before revisions but also after the revisions (in the follow-up period) compared to those without trauma history. An interesting and recently proposed theory, “Trojan Horse” hypothesis, posits that the bacteria translocation is not necessarily mediated by blood, such as neutrophils and macrophages that can act as Trojan horses to transfer pathogens [13–15]. The local immune cells can engulf the proliferating pathogens and co-exist with the pathogens. Then, the pathogen-carrying immune cells enter the circulation and travel to susceptible tissues, releasing infectious payload. According to this theory, the immune cells in the body can transfer pathogens from remote sites, such as the teeth, gums, or gastrointestinal tracts to the injured periprosthetic environment, where they unload pathogens and cause PJI [15–18]. In patients with trauma, the immune cells and the pathogens are also more likely to penetrate the vessel barrier because of the increased permeability of vessel walls [16, 19, 20]. The immune cells can be aggerated in the injured sites to eliminate the necrotic tissues when trauma occurs to the periprosthetic tissues by which pathogens carried by the immune cells may colonize the prosthesis, increasing the risk of PJI and causing PJI when trauma happens to the joint after TJA. The “Trojan Horse” hypothesis of trauma-associated periprosthetic joint infection is shown in Fig. 3. We believe that the “Trojan Horse theory” can partially explain the relatively higher rate of infection observed in revision patients with trauma history but concrete clinical evidence and prospectively microbiological study are still warranted to further support the hypothesis in PJI.

The patients with trauma history before revisions also carried a higher rate of aseptic failure such as mechanical complications, periprosthetic joint fracture, aseptic loosening, and abnormal incision healing. Prior trauma of the involved joint may indicate impaired motor abilities, followed by the trauma after revisions, thereby leading to a relatively higher risk of mechanical complications and fractures. The relationship between these pathogen-engulfing immune cells and surgical site infection (SSI) has been revealed in rat model but the association between pathogen-carrying immune cells and abnormal incision healing in joint revisions still needs to be explored further in the future studies [15]. Moreover, several studies also demonstrated that some patients classified as aseptic loosening may actually be the cases of infection in origin but were not diagnosed as PJI against the available criteria [8, 10, 21]. Using molecular diagnostic methods, some pathogens were also found in the aforementioned aseptic cases [22]. Therefore, it is plausible that some of these aseptic complications in these patients with trauma history after revisions may also represent/be treatment failure as a result of infection, and pathogens may play a partial role in the process. In recent years, the association between impaired colonization niche and surgical site infection was explored extensively but it remains “gray” in the field of total joint arthroplasty [13, 16, 19]. And the influence of the dysbiosis of colonization niche (gut, gum, lung) on the outcomes of total joint arthroplasty in PJI and non-PJI patients will be a popular topic in the future.

This study still had several limitations. Firstly, the sample size of patients with trauma was relatively small, which may subject the study to a higher risk for type-two error, and multi-center studies are necessary to further confirm the results of this study. However, with the given sample size, significantly different primary and secondary outcomes between the patients with trauma and patients without trauma were detected in this study. Moreover, this study was performed in a tertiary joint center retrospectively and thus had some inherent limitations such as recall bias and selection bias. Secondly, we used the Trojan Horse hypothesis to interpret why patients with trauma before revisions were more likely to suffer from PJI compared to those without trauma history, but no extensive study was conducted to verify the proposed mechanisms and the association between injured joint and the microorganisms of colonization niche (gum, gut, respiratory tract) because of the retrospective design of the study. In the ensuing studies, we will prospectively examine the mechanisms and the influence of these colonization niches on the injured joints after total joint arthroplasty. Thirdly, in the tertiary health-care center for PJI, the infection/PJI rate after revisions was high because patients highly suspected of PJI were admitted. Besides, only knees and hips were included in this study and no other joints were evaluated in this study. This retrospective nature of the study can also introduce some additional bias and further multi-center studies involving other joints are necessary. Finally, the data about the degree of trauma weren’t evaluated in detail because of the retrospective design of the study, and following prospective study which includes detailed imaging information on trauma degrees, such as microbiota in the colonization niche, MRI, PET and SPECT can offer quantitative support to the hypothesis.

Despite the limitations, our study demonstrated that patients with trauma history before revisions were at higher risk of PJI compared to those without and potentially provided the clinical evidence supporting Trojan Horse hypothesis that explains the development of periprosthetic joint infection. Moreover, a minimal 3-years of follow-up revealed that the patients with trauma carried higher risk for treatment failure due to PJI, aseptic loosening and other complications after the revisions. Therefore, we believe that, when periprosthetic joint fracture develops, revision patients with trauma should be given special medical attention or care to increase the treatment success rate.

## Conclusions

Our study demonstrated that revision patients with trauma history carry higher risk of PJI compared to those without. Moreover, after revisions, the trauma patients were still at higher risk for treatment failure due to PJI, periprosthetic joint fracture and mechanical complications.

### Supplementary Information

**Additional file 1.**

**Additional file 2: Table S1. **The trauma history of patients in the trauma cohort.

## Data Availability

All data and materials were in full compliance with the journal’s policy. The data were obtained from the Department of Orthopedic Surgery, The First Medical Center, Chinese PLA General Hospital. The datasets used during the current study are available from the corresponding author upon reasonable request.

## Abbreviations

TJA: Total joint arthroplasty
PJI: Periprosthetic joint infection
PSM: Propensity scores matched
