# Supplementary material for: Increased risk of periprosthetic joint infection after traumatic injury in joint revision patients
Source: Arthroplasty. 2024 Feb 5;6:8. doi: 10.1186/s42836-024-00235-5 (PMC10840204; doi:10.1186/s42836-024-00235-5)
Supplement: Supplementary file 2 — Additional file 2: Table S1. The trauma history of patients in the trauma cohort. [file 42836_2024_235_MOESM2_ESM.docx]

***Appendix table1: The trauma history of patients in the trauma cohort:***

| Location | Number, n(%) | Interval between trauma and revisions (months) | Number, n(%) | Severity | Number, n(%) |
| --- | --- | --- | --- | --- | --- |
| Hip | 43,60.56% | 0-1 | 40,56.34% | periprosthetic joint fracture | 29,40.85% |
| knee | 28,39.44% | 1-2 | 15,21.27% | wrench injury | 20,28.17% |
|  |  | 2-3 | 16,22.54% | tumble injury | 22,30.99% |
